# The development of national multisectoral action plans for the prevention and control of noncommunicable diseases: experiences of national-level stakeholders in four countries

**DOI:** 10.1080/16549716.2018.1532632

**Published:** 2018-11-13

**Authors:** Kremlin Wickramasinghe, Elizabeth Wilkins, Charlie Foster, Ibtihal Fadhil, Asmus Hammerich, Slim Slama, Hicham El Berri, Manal Elimam, Salim Adib, Mona Al-Mudwahi, Nick Townsend

**Affiliations:** a World Health Organization European Office for Prevention and Control of Noncommunicable Diseases, Noncommunicable Disease Office, Moscow, Russian Federation; b Centre on Population Approaches for NCD Prevention, Nuffield Department of Population Health (NDPH), University of Oxford, Oxford, UK; c Centre for Exercise, Nutrition and Health Sciences, School for Policy Studies, University of Bristol, Bristol, UK; d Department of Noncommunicable Diseases and Mental Health, World Health Organization (WHO) Regional Office for the Eastern Mediterranean (EMRO), Cairo, Egypt; e Non communicable Diseases Division, Epidemiology and control Diseases Directorate, Ministry of Health, Rabat, Morocco; f Non communicable Disease Division, Primary Health Care Directorate, Federal Ministry of Health, Khartoum, Sudan; g Department of Epidemiology and Population Health, American University of Beirut, Beirut, Lebanon; h World Health Organization Liaison Office for Somalia, Somalia WHO Country Office, Nairobi, Kenya; i Department for Health, University of Bath, Bath, UK

**Keywords:** Multisectoral, noncommunicable disease, prevention, policy

## Abstract

**Background**: In October 2012, the WHO Eastern Mediterranean Region (EMR) developed a Regional Framework for Action to implement multisectoral action plans (MAPs) for the prevention and control of noncommunicable diseases (NCDs).

**Objectives**: The aim of this project was to draw on the experiences of four EMR countries that had made good progress in developing these MAPs, to identify best practice and barriers in the development of them.

**Methods**: Structured interviews were held with key stakeholders in the development of the MAPs from the four focal EMR countries: Lebanon, Morocco, Sudan, and Yemen. These interviews comprised two stages: first we conducted face-to-face interviews in September 2014; we then carried out follow-up teleconference interviews during October 2014. Thematic analysis of transcripts was used to identify several themes, including examples of best practices and challenges that were common to all four focal countries and are likely to be also relevant to many other countries in the development of MAPs.

**Results**: Best practice in the development of MAPs includes methods to identify and recruit key sectors, ways to foster collaboration between sectors in the development and implementation of the action plan and means through which to encourage public support. Challenges identified included measuring outcomes in evaluating MAP success, current pressures and competing priorities for sectors and the perception of health issues as the responsibility of the health sector. Cultural and bureaucratic challenges were also discussed along with multisectoral fatigue, through the promotion of multisectoral approaches for a number of national issues.

**Conclusions**: Although the development of multisectoral action plans to tackle NCDs is recommended, the process is a challenging one. Reflections from those countries which have experience in developing such action plans is important in identifying common challenges as well as recommending best practice, such that other countries may learn from their experiences.

## Background

Noncommunicable diseases (NCDs) represent a huge and growing burden globally, posing significant challenges in both high-income and low- and middle-income countries[1]. The United Nations (UN) General Assembly 1st High Level Meeting in 2011 received the commitment from World Leaders to take measures to tackle NCDs [2]. Since then there have been various policy interventions and programmes to support this agenda, including the inclusion of NCDs with measurable targets and indicators under the third of the Sustainable Development Goals (SDGs) in 2015 [3].

Within the 2nd High Level Meeting of the UN General Assembly on the Comprehensive Review and Assessment of the Progress Achieved in the Prevention and Control of NCDs in 2014, Member States agreed to four time-bound commitments to be achieved by 2015/2016: 1) Consider setting national targets for 2025, 2) Consider developing or strengthening national multisectoral policies and plans (MSAPs), 3) Reduce risk factors for NCDs and underlying social determinants through the implementation of interventions and policy options to create health-promoting environments, and 4) Strengthen and orient health systems to address the prevention and control of NCDs and the underlying social determinants. The World Health Organization (WHO) will report on the progress in achieving these commitments to the 3rd UN General Assembly High Level Meeting in 2018 [4].

The second of these commitments, to develop national multisectoral policies and plans to achieve the national NCD targets, recognises that many of the drivers of NCDs and their risk factors lie outside the control of the national health sectors [5]. A successful and sustainable strategy to tackle NCDs therefore requires the collaboration of all of these sectors and stakeholders [6,7], i.e. a multisectoral approach; usually defined as either ‘whole-of-government’ or ‘whole-of-society’ action [1,8] .

Many countries have started the process of developing and implementing MSAPs [7,9–14] with technical support provided by the WHO [15] through recommended processes [16]. However, this support, along with the existing literature on multisectoral working, has been developed either conceptually or through expert opinion [1,5,15]. It does not consider the practical implications countries may encounter when they attempt to follow the recommended steps.

This is not a traditional area of expertise for the Ministries of Health in most countries. In many countries Ministries of Health are not aware of the various practical challenges they may encounter in developing MSAPs and the solutions that may help them overcome them. Countries and their national-level stakeholders need careful guidance and support, yet the current literature does not provide any guidelines on, or examples of how to, develop MSAPs based on the real experience of national-level stakeholders.

The WHO Eastern Mediterranean Regional Office (EMRO), based in Cairo, Egypt initiated a process to support countries to develop MSAPs in their region [17] as an activity under the WHO EMRO regional action plan [18], following the 2011 UN Political Declaration on NCDs. In the first phase, WHO EMRO supported four selected countries to develop MSAPs. This study aimed to identify the common stages these four countries followed to develop MSAPs, the challenges they faced in doing so and possible solutions they identified, with a view to their experiences providing practical guidance to other countries that are currently developing MSAPs.

## Methods

Between August and December 2014, we interviewed national NCD directors from Ministries of Health, other representatives from ministries, WHO members and lead academic experts, all of whom had led the national MSAP development in the four countries: Lebanon, Morocco, Sudan, and Yemen. These were the first four countries to start developing NCD MSAPs with the support of WHO EMRO. Recruitment occurred through the WHO-EMRO office, which contacted national NCD directors in all countries, all of whom agreed to take part in the study.

All interviews were conducted with two interviewers. CF and NT carried out face-to-face interviews with representatives from each country in Cairo in September 2014 when they attended the regional NCD progress review meeting. In October and November 2014, EW and KW carried out follow-up teleconference interviews from the UK, with the same representatives and other relevant individuals who could not attend the meeting in Cairo. A total of 14 individuals were interviewed for this study. All interviews were carried out in English. A translator was offered for all participants, should they want one, although this was used in one face-to-face interview only.

All interviews were semi-structured but followed an interview template to allow for consistency. The objective of the face-to-face interviews was to investigate the process followed, including key stages, in developing the MSAPs. The template was designed to reflect this aim. The template for the telephone interviews was designed to investigate themes of barriers and facilitators around each of these stages. The semi-structured nature of the interviews allowed participants to introduce topics and themes not covered in the templates.

The first interview aimed to identify key processes that participants had gone through and their reflections on these. As this was exploratory work on the process, which was largely unknown before the interview, we used content analysis to identify key stages of MSAP development that were common to all participants. The aim of the follow-up telephone interviews was to explore, in more depth, the perception of participants of barriers and facilitators for each stage. Thematic analysis was used to identify themes on these that occurred within the stages identified in the first round of interviews.

We used a combination of deductive and inductive analysis. The initial identification and coding of stages was inductive, following those identified by participants in the first interviews. These stages were used as broad codes in the second round of interviews, leading to a more deductive approach. However, within these stages we also used an inductive approach as participants identified themes related to barriers and facilitators at each stage.

These methods allowed for investigator triangulation, by using two interviewers at each interview stage and different interviewers between the data collections. The collection of data from more than one participant from each country allowed for data source triangulation.

## Results

**Figure 1.**
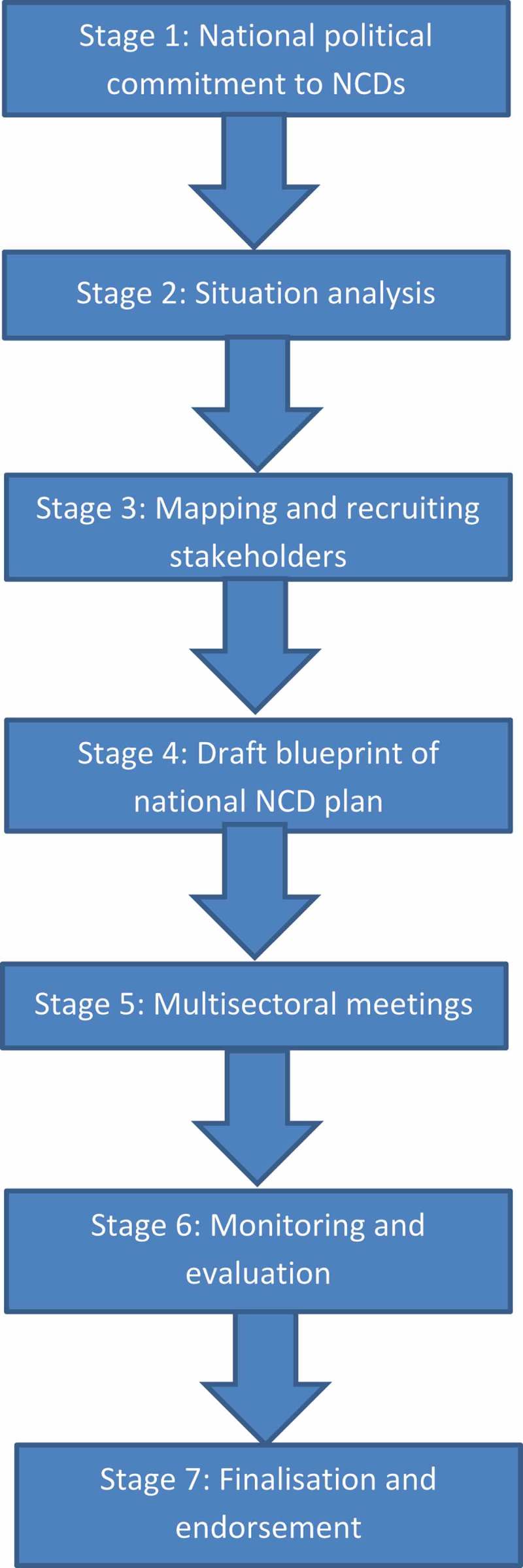
Stages in the process of developing a multisectoral action plan to address noncommunicable diseases.

We identified a number of common stages completed by countries in the development of multisectoral action plans (MSAPs) (Figure 1).

At each stage of this process we identified best practices, recommendations and common barriers countries faced:

**Step 1) National political commitment to NCDs**

Participants reported that country leaders must recognise NCDs as a major problem and commit to devoting physical, human, and time resources to address these diseases if MSAPs are to be successful. Participants highlighted that the personal experience of senior policy makers with NCDs was helpful in generating strong political support.

*‘Senior support in the ministries is one of the key issues …. we need a high level inter-ministerial committee to support this, to advocate, to give guidance to the technical departments in the ministries and so on…’*

*National NCD Director, Country C*

They reported that the involvement of the WHO in this process enabled them, in many instances, to get the attention and support from the political leaders. However, it was felt that they needed to make it clear to policy makers that the WHO would not provide financial support and there was a reported lack of adequate resources committed to the development of MSAPs.

*‘They always think WHO is going to fund, and we’re always trying to show WHO support is in terms of technical, with guidelines and policies and standards and it’s not really to do things at ground level.’*

*County level WHO officer, Country B*

Participants recommended that one of the first outcomes of a national political commitment to NCDs is the establishment of a high-level national committee and specific NCD unit within the government’s Ministry of Health. A common challenge identified by participants, however, was in managing conflicts of interests at this stage. Some political leaders owned businesses or shares in companies, which may be affected by policies to combat NCDs and their risk factors. In addition, some countries faced long-term political instability, with participants reporting that it was difficult to generate policies, laws or commitment for MSAPs in these circumstances.

*‘We have an anti-smoking law in our country. … there is a conflict of interest with some politicians … some of the people are the owners of restaurants who are really pushing against this law’ .*

*Public Health Academic, Country D*

**Step 2) Situation analysis**

A situation analysis would commonly involve the collection of country-specific NCD-related data in terms of mortality, morbidity, risk factor prevalence, and economic and social impact. Participants suggested that these data would provide an indication of the size of the NCD burden and hence the extent of the action required. The findings from the situational analysis could also then be used to set national targets and tailor MSAPs to country needs by highlighting specific diseases and risk factors that are particularly relevant in the national context. In addition, although most countries reported that this step followed securing political commitment, data from the situation analysis also proved helpful in generating political support.

‘We used the data in the household survey … for example hypertension was twenty-four per cent of the population. So the policymakers themselves realise that it’s a big problem.’

*NCD officer, Country D*

These data, however, are not regularly collected nor available in all countries and participants found that not having regular up to date statistics was a major challenge, as it compromises their decision making process and targeting of an MSAP. Although it was reported that the WHO was providing technical assistance in some countries, it was also recognised that national public health agencies were busy with other traditional work programmes, such as maternal and child health or infectious diseases. Therefore, they did not always have the capacity to conduct these NCD related surveys.

It was also suggested that where these data were collected, gaining an accurate picture of the NCD burden was challenging. This was specifically the case in this region; due to cultural issues that made it very difficult to collect data on some NCD related behavioural risk factors, such as alcohol intake.

‘most surveys are conducted through the primary healthcare centres, some of them are in conservative areas …. even if you drink you will not admit that you are drinking’.

*NCD officer, Country C*

Although all countries mentioned the utility of WHO STEPwise surveys in the collection of data, three also discussed regional inequalities in data coverage within their countries.

**Step 3) Mapping and recruiting stakeholders**

Before an MSAP can be constructed, it is necessary to identify and recruit the relevant sectors and stakeholders. Participants described various starting points they had used to identify potentially relevant stakeholders including WHO guidance on relevant stakeholders for multisectoral action to address NCDs, employment of an external consultant to devise an initial list of potentially relevant stakeholders and identification of stakeholders already involved in Primary Health Care multisectoral collaborations.

Participants from all countries first identified relevant individuals within the Ministry of Health. They then identified a mixture of non-health government sectors and non-government stakeholders who should be involved in their national NCD action plans including Youth and Sport, Education, Higher Education, Trade, Finance and Agriculture and the Environment.

Participants reported that they had identified a number of relevant non-governmental organisations (NGOs), including those involved with general health and those involved with specific chronic diseases. These included UN agencies (such as the country-specific WHO office, UNICEF, the United Nations Population Fund (UNFPA)) and academic experts, members of the pharmaceutical and food industries, along with external consultants/experts and civil society groups. Following the initial brainstorming of relevant sectors and stakeholders, one country categorised these partners into two groups according to the level of priority. They also described a number of already established collaborations that helped them to identify and reach existing stakeholders, these included collaboration between the Ministry of Health and the Ministry of Education, along with links between Primary Health Care units and grassroot-level NGOs and other organisations.

Having identified relevant stakeholders, participants identified several factors which contributed to their successful recruitment. Initially they identified specific individuals or focal points in the relevant sectors. Sending invitations to these individuals from senior officials in the Ministry of Health was seen as important in encouraging their involvement.

‘we write a letter, signed by our General Secretary, and he sent it to their General Secretaries. That’s maybe the thing, maybe the main thing why these people were there.’

*National NCD Director, Country D*

Highlighting existing policies of stakeholders and recognising their contribution to NCD health, was recognised as an excellent platform to strengthen partnerships and recruit stakeholders for the MSAP development process. It was felt that asking them to commit to something seen to be new would be discouraging and participants found that mapping the existing contributions of stakeholders and highlighting them in the initial discussions was beneficial in encouraging them to commit to it.

*‘Individuals from the Ministry of Health explained to members of the Ministry of Education, that their policies to promote a healthy diet and to increase physical activity within the school curriculum are having a positive impact in tackling NCDs. This in turn encouraged the Ministry of Education to get involved in the project.’*

*National NCD Director, Country A*

A number of challenges at this stage were also identified. Most sectors and stakeholders had planned annual activities within their own departments and ministries, making it difficult for them to attend regular meetings organised to develop the NCD MSAP. In addition, in some countries there was a pervading general/cultural view that NCDs are destiny, with non-health sectors also often viewing NCDs as individual responsibility. Stakeholders therefore struggle to identify the role of their sectors in the prevention and control of NCDs. In addition, multisectoral policies and programmes have been developed in a number of other areas, such as environmental sustainability and social care. As the same senior officials get invited to all these meetings ‘multisectoral fatigue’ was identified as a major challenge in recruiting them for an NCD MSAP. Even when sectors have committed their support at meetings, participants sometimes found that it was difficult to maintain the continuity of discussions as the main contact point in these sectors can change.

*‘you go to sectors, you meet people, next time you’re meeting other people … someone comes, very engaged and so on, he goes, next meeting someone else who is not so engaged and not so interested comes.’*

*NCD officer, Country C*

**Step 4) Drafting a blueprint of the national NCD action plan**

Developing a blueprint, or outline, of the NCD MSAP, which will subsequently undergo revision, was recognised as key to the production of a final action plan. All participants identified that stakeholders should be consulted as early as possible during the initial phases, to achieve a successful blueprint of the action plan. In all four countries this stage has taken place before the first multisectoral consultation, and in one country it was conducted even earlier, before the mapping and recruitment of stakeholders.

*‘Right from the start of the project … it is important to integrate other participating sectors fully. From the first multisectoral meeting, the attending sectors were encouraged to propose their own ideas for policies to address NCDs. Since then, there has been frequent open contact between the NCD unit and the members of other sectors.’*

*National NCD Director, Country C*

In countries with dedicated NCD units, these units took responsibility for developing the outline plan. Common themes in the outline of MSAPs in countries included: (1) Raising the priority of NCDs at all levels of society, (2) Reducing exposure of the population to the shared modifiable NCD risk factors, (3) Strengthening and reforming health systems to improve the control of NCDs, and (4) monitoring and evaluation of NCDs and their impacts. NCD targets from global documents, such as the nine global voluntary targets of the Global Action Plan for the prevention and control of NCDs were included, although participants reported that targets from this and other WHO documents could be challenging if this conflicted with cultural beliefs. They also noted the challenge of developing an action plan that balanced the nine voluntary targets but that was appropriate and achievable in their national context.

Participants highlighted the importance of prioritisation in developing the MSAP, although some countries felt they didn’t have adequate data and tools to enable them to do this. In addition, although MSAPs were developed for a national approach, it was recognised that these would need to be adapted for regional action in the country. In order to achieve this, participants recognised the importance of community involvement at this stage of the process.

**Step 5) Multisectoral meetings**

The organisation of multisectoral meetings or workshops was seen to be critical in the development of a national NCD MSAP. These meetings started early in the process with many relevant sectors and stakeholders brought together to review the drafted blueprint of the action plan and to agree on their specific tasks, roles, and responsibilities. Several such multisectoral meetings will typically be conducted, each with a clear agenda (e.g. introduction to NCDs and their risk factors; brainstorming of policy ideas; advocacy) throughout the development of the MSAP. Between each meeting, re-drafting of the action plan takes place, with the new draft being presented for discussion at the next meeting. Accordingly, this stage of the process can be quite lengthy, with time frames exceeding one year.

In all four countries, the sectors and stakeholders in attendance at the multisectoral meetings seem to have been very willing to make commitments that will help in the prevention and control of NCDs. Participants mentioned that it is important to remember, however, that this is only the planning stage; evidence about the need for negotiation in the implementation stage, which was yet to come in each of the four countries, is not yet available. Although these meetings were seen to play an important role in developing MSAPs, a common challenge identified by participants was the logistical difficulty in arranging a meeting with representatives from several sectors, due to conflicting commitments for each stakeholder. Due to these logistical issues, some NCD directors decided to follow up with each sector separately.

‘Okay, due to this problem, after that, I decided to go to each sector on their own place. We divide ourselves (in)to many teams and go to them and have our discussion’

*National NCD Director, Country B*

**Step 6) Monitoring and evaluation**

Monitoring and evaluation of both the ‘multisectoral’ element of the project (‘how effective the engagement of other sectors has been’) and of the action plan itself (‘to what extent the targets set out in the action plan have been reached’) was seen as important. Participants recognised that the monitoring and evaluation plans should be included in development of the MSAP itself and that this step needed further technical support from WHO and other experts. They felt that developing indicators to measure some MSAP activities was challenging, highlighting the lack of agreed scientific tools for this purpose of measuring ‘multisectoral involvement’ despite its importance.

In other instances, although reliable indicators had been identified, cost was an issue in implementing them. Participants reported that they would drop more expensive data collection from their monitoring and evaluation framework, as their inclusion would make it very difficult to complete the task. WHO STEPwise surveys were again mentioned as a useful source for monitoring data.

‘to measure the salt content in the urine for example, … doing some sort of twenty-four hour urine (test), is a very difficult process.’

*NCD officer, Country A*

**Step 7) Finalisation and endorsement**

Participants from all countries recommended that finalisation and endorsement should not be delayed as stakeholders and communities that had been involved in the development of the MSAP could lose interest, leading to a loss of momentum. However, it was recognised that this was a lengthy process due to the involvement of different sectors on different topics, with delays found in obtaining agreement from all stakeholders. NCD teams were then required to balance these challenges against targets which could include time-bound deadlines.

*‘They were saying we have to finish by June, and at the same time they’re saying we have to consult widely with the sectors … we will have a document submitted by June but consider it a dynamic document’’*

*National NCD Director, Country D*

## Summary of findings

These stages, along with the challenges (Table 1), best practice and facilitators identified (Table 2), focused on the development of an MSAP. Participants from all countries mentioned that the development of such policies, although important, was the initial stage and that challenges beyond the development of an MSAP would arise when countries began to implement it. Recommending continued evaluation of this process and the need for continued WHO support.

**Table 1.** Common challenges in MSAP development.

Stage	Common challenges
1) National political commitment to NCDs	Political instability
	Lack of understanding of roles of non-health sector for NCD prevention and control
	National-level leaders and other stakeholders expect WHO to finance the action plan
2) Situational analysis	Sub-optimal surveillance data
	Competing priorities at national level
	Difficult to obtain reliable data on sensitive issues such as alcohol consumption
3) Mapping and recruiting stakeholders	NCDs are viewed as a health sector issue
	Cultural challenges- common belief that health is destiny
	A bureaucratic process
	Multisectoral fatigue
	Lack of continuity in participation from other sectors
4) Drafting a blueprint of the national NCD plan	Selecting relevant targets
	Lack of tools and methods to prioritise targets
5) Multisectoral meetings	Logistical challenge of bringing multiple sectors together
6) Monitoring and evaluation (M & E)	Not having tools to monitor and measure ‘multisectoral component’
	Lack of technical expertise to develop M & E plan at national level
	Lack of resources, even when methods are available
7) Finalisation and endorsement	Lack of continuity from non-health sector participation
	Different officers come to meetings and some of them cannot make decisions

**Table 2.** Recommended best practices and facilitators in MSAP development.

Stage	‘Best practices and facilitators’
1) National political commitment to NCDs	Send letters from senior officers
	Provide person experience related to the burden of NCDs and explain their determinants
	Clarification at early stage that WHO provides technical support and identify required resources as part of the process
	Showing the leadership and support from WHO, encourages other sectors to respond positively
2) Situational analysis	Use existing survey tools and build the capacity to collect good reliable data
3) Mapping and recruiting stakeholders	Ensure effective collaboration within the Ministry of Health before reaching out to engage other sectors
	Conduct some sort of stakeholder analysis and categorise them according to the relevance
	Send invitations from high-level officials
	Select a specific focal point in each sector
	Emphasise current contributions of non-health stakeholders as a starting point
4) Drafting a blueprint of the national NCD plan	Fully involve other sectors in the development
	Enhance community engagement in the process
	Consider adoption of the plan from national to regional
5) Multisectoral meetings	Develop shared understanding
	Follow-up meetings could be with individual sectors
6) Monitoring and evaluation (M & E)	Map available resources before finalising the M & E plan
	Define measurable targets
7) Finalisation and endorsement	Formally agree the roles and responsibilities
	Document may change; consider it as a dynamic document.
	Clearly state that Ministry of Health would lead the process

## Discussion

This study was conducted among national-level stakeholders from four countries in the WHO EMR, who played a key role in developing their national NCD MSAPs. Findings are based on the responses provided by these participants. A major limitation of the study is that we only obtained data from the key individuals who led the process in each country. We did not, therefore, provide views from the range of non-health sectors involved in the action plan development. Despite access to the key personnel in the development of national MSAPs being a strength in providing guidance for countries that are trying to do the same, there may be some subjectivity to their views.

Previously published guidelines have highlighted similar steps in the development of NCD MSAPs [5]. The WHO tool box [15] which provides technical assistance to develop national NCD MSAPs includes a number of different stages of development, but this guidance also includes implementation and evaluation stages, which the current paper does not. Additionally, whereas this study gained evidence from stakeholders who had experience in developing MSAPs, the WHO tool box was developed from expert opinion rather than country-level experience.

Strong leadership by governments in tackling NCDs is one of the first points highlighted by experts [19]. Since the UN high-level meeting on NCDs in 2011, governments have started showing the political leadership to tackle NCDs by adopting the NCD MSAPs [20]. Although evaluation of the MSAPs was considered crucial by all the participants in this study, none of them have so far tried to evaluate ‘multisectoral involvement’. This is a challenge as countries do not have agreed methods or capacity to do so, whilst the current literature does not provide any specific methods or tools to measure this component. Developing a method to measure ‘multisectoral involvement’ is one of the most important steps required to generate evidence and to support MSAP development. Although there were several common views expressed by the four countries we recruited for this study, there was also variation. This emphasises the fact that a general guidance framework can be really useful for countries, but at the same time countries need to be mindful of adapting it to their own context to ensure best possible outcomes. This study also identified a projected need for further research to identify best practices and challenges in the later implementation stages of the MSAP.

## Conclusion

National MSAP development may be seen as challenging and is not a capacity regularly and routinely available in the Ministry of Health, but it could be achieved through collaboration and good technical support. The involvement of different stakeholders from the beginning, along with support from WHO and academics is recognised as important. Sharing of case studies, which highlight common challenges and best practices, has the potential to support the process of MSAP development in countries. Availability of up-to-date, relevant data, access to technical support for areas such as prioritisation of actions, costing the action plan and monitoring and evaluation methods would significantly improve the process of national NCD MSAP development. Regular opportunities to share national experience will help the countries to achieve this time-bound target of having an operational NCD MSAP and contribute to the NCD related SDG targets by 2030.

## Paper context

WHO Member States agreed to consider developing, or strengthening, national multisectoral policies or plans for the prevention and control of noncommunicable diseases. Reviewing this process with those tasked to do it is important, as past guidance was developed conceptually. This study worked with the first four countries to develop MSAPs in the WHO Eastern Mediterranean Region, to identify key stages the countries went through, the challenges they faced and possible solutions they identified.
